# Effect of Tillage Treatment on the Diversity of Soil Arbuscular Mycorrhizal Fungal and Soil Aggregate-Associated Carbon Content

**DOI:** 10.3389/fmicb.2018.02986

**Published:** 2018-12-06

**Authors:** Xingli Lu, Xingneng Lu, Yuncheng Liao

**Affiliations:** ^1^College of Agronomy, Ningxia University, Yinchuan, China; ^2^Yinchuan Provincial Sub-branch, The People’s Bank of China, Yinchuan, China; ^3^College of Agronomy, Northwest A&F University, Yangling, China

**Keywords:** AM fungi, no-tillage, soil carbon, maize field, Loess Plateau

## Abstract

No-tillage agriculture can sustain productivity and protect the environment. A comprehensive understanding of soil arbuscular mycorrhizal (AM) fungal diversity and soil carbon distribution within aggregate fractions is essential to the evaluation of no-tillage agriculture. The long-term field experiment included two tillage treatments (1) no tillage with straw returned to the soil (NTS), and (2) conventional mouldboard-plowing tillage without straw (CT), and was conducted on the Loess Plateau, north-western China, from October 2009. The soil samples were collected from the surface layer (0–20 cm depth) at the maturation stage of the summer maize (*Zea mays* L.) for analyzing aggregates separated by the dry-sieving method. The organic carbon content in the bulk soil and different particle size aggregates were measured using the dichromate oxidization method. The species compositions of soil AM fungi were compared by applying high-throughput sequencing of 18S rRNA. The results showed that the NTS had 9.1–12.2% higher percentage of soil macro-aggregates, resulting in 9.8% increase in mean weight diameter and 10.0% increase in bulk soil organic carbon content as compared with CT treatment. In addition, the NTS treatment had significantly higher percentages of *Septoglomus* and *Glomus* than the CT treatment. We also found some significant differences in the fungal communities of the soils of the two treatments. There was a strong positive relationship between bulk soil organic carbon and the percentages of *Septoglomus* and *Glomus*. Our results suggested that the NTS treatment had a protective effect on AM fungal community structures, which might play a key role in the development of agricultural sustainability in the Loess Plateau of China.

## Introduction

With increasing global interest in climate change, there has been increasing interest in the potential for carbon (C) sequestration in agricultural soil. Soil is not only the basis of crop production but is also the key facilitator of C sequestration in terrestrial ecosystems. As the largest C pool in the terrestrial ecosystem, soil has high ecological value. Carbon dioxide (CO_2_) emissions from soil play a key role in C balance at the continental scale. At the global scale, the top 1 m layer of soil contains about 1,500 Pg of soil organic carbon (SOC; Song et al., 2016). The accumulation of SOC is considered to be the best choice for long-term C sequestration in the terrestrial ecosystem. Moreover, SOC and CO_2_ can be mutually converted. Once a soil ecosystem is destroyed, the rate of decomposition of organic carbon in the soil will be accelerated, greenhouse gas emissions will increase, and the greenhouse effect will be intensified, resulting in global warming (Zhu et al., 2016; Forte et al., 2017; Krauss et al., 2017).

In addition, soil aggregate stability and SOC are important indicators of soil quality and environmental sustainability in agro-ecosystems. Firstly, the decomposition and transformation of SOC are affected by aggregation construction (Zhang et al., 2008; Zhao et al., 2015, 2018). It has been reported that stable aggregates can physically prevent SOC against rapid decomposition (Sun et al., 2018). Secondly, SOC is considered to be the main binding agent contributing to aggregate stability (Chenu et al., 2000; Bhattacharyya et al., 2011). Moreover, the SOC content in macro-aggregates is an important index of soil aggregate stability and C loss, as influenced by various management methods (Sheehy et al., 2015). For example, the quantity and stabilization mechanisms of SOC, as related to soil aggregates, are influenced by tillage practices (Crittenden et al., 2015). Previous studies have shown that unreasonable tillage will destroy the stability of a soil ecosystem over the long term, including its soil aggregate stability, soil porosity and soil nutrients, causing a series of changes in the soil’s physical and chemical characteristics (Bartz et al., 2014; Crittenden et al., 2015; Buchi et al., 2017). Extensive experiments have reported that conservation tillage, such as the adoption of a no-tillage regime, can increase soil macro-aggregates formation and stability, and offer a good protective effect for SOC (Mikha et al., 2013; Kumar et al., 2014; Dai et al., 2015), as compared with conventional tillage systems.

The total area of the Loess Plateau, China, is 640,000 ha and soil erosion affects 60.9% of it. Thus, soil erosion is one of the main environmental problems on the Loess Plateau (Huang et al., 2002). Wang et al. (2015) reported that one of the main causes of soil erosion on the Loess Plateau is the adoption of irrational management measures. Traditional tillage not only increases soil erosion but has also led to a continuous reduction in soil fertility by removing large amounts of crop straw, which is associated with great mechanical disturbance (Zhang et al., 2016). Arbuscular mycorrhizal (AM) fungi have an important role in biogeochemical cycles and contribute to many terrestrial ecosystem functions (Harley and Smith, 1983; Piotrowski et al., 2004; Wang et al., 2018). For example, AM fungi have a vital role in improving crop growth and enhancing crop resistance to plant disease (Rillig, 2004). Furthermore, the formation and conservation of soil aggregates are always influenced by the extra-radical hyphae of AM fungi (Rillig et al., 2010; Dai et al., 2015). The community composition of AM fungi is also easily influenced by variations in land-use types and agricultural management methods (Martinez and Johnson, 2010; Xiang et al., 2014; Zhao et al., 2015). Thus, there is concern about the responsible conservation of AM fungi in cultivated fields for sustainable crop management. However, there is limited systematic information pertinent to soil aggregates, their associated C content and AM fungal diversity under different tillage regimes on the Loess Plateau.

Thus, to estimate the impacts of no-tillage with straw return (NTS) practices on AM fungal diversity, bulk SOC, and soil aggregates and their associated C contents, and reveal the main factors that affect SOC in bulk soil in relation to conventional mouldboard plowing tillage without straw (CT), a long-term experiment applying high-throughput sequencing of 18S rRNA was conducted in the Loess Plateau, China. We hypothesized that tillage regimes influence AM fungal community composition via alteration of soil physical and chemical characteristics, which ultimately influence bulk SOC. The objectives of the present study were to: (i) explore the changes in AM fungal community composition after 7 years of no-tillage, and (ii) study the relationships between these changes and soil physical and chemical characteristics, such as soil aggregate composition, associated C contents, and bulk SOC, to determine the key factors influencing bulk SOC.

## Materials and Methods

### Site

The experiment was conducted at the Northwest A&F University farm (latitude 34°21′ N, longitude 108°10′ E). The experiment included two tillage treatments: (1) no tillage with crop straw return (NTS) and (2) conventional tillage without crop straw (CT). The same tillage treatments were used over 7 years (2009–2015) in plots measuring 18.3 m × 15 m. The two tillage treatments were designed in a randomized block and included three replications. The crop system was a winter wheat (*Triticum aestivum* L.)-summer maize (*Zea mays* L.) rotation system.

The fields were cultivated twice: once after harvesting winter wheat in June and once after the summer maize harvest in October. The field was plowed to 20–25 cm depth in the CT treatment, and then a rotavator was applied to plow the soil to 15 cm depth. No-tillage machinery disturbed the soils in the NTS plots either before or after the establishment of the trial, except during sowing when a no-tillage planter was used.

### Soil DNA Extraction

At the maize harvesting stage (October, 2015) three replicates of soil samples from the NTS and CT treatments were selected. The soil specimens were taken from 20 points at surface depth (0–20 cm) for each plot. Samples were mixed and sieved through a 2 mm square aperture mesh to remove stones and plant material (including above-ground materials and roots) and kept at -80°C until analysis. Microbial DNA was taken from 0.25 g of fresh soil by applying a TIANamp soil DNA kit according to the manufacturer’s instructions. The A260/280 ratio and agarose gel electrophoresis were applied to control the DNA quality and integrity. The genomic DNA was kept at -20°C until PCR amplification and metagenomic sequencing were conducted.

### PCR Amplification and Preparation of the Amplicon Libraries

PCR amplification was performed in a GeneAmp PCR System 9700 (Life Technologies, Carlsbad, CA, United States). The hypervariable regions (V3–V4) of 18S rDNA were used to distinguish the species of fungi. We synthesized the primers based on the changeable region of V3–V4 (F: 5′- GCCTCCCTCGCGCCATCAG -3′, R:5′-GCCTTGCCAGCCCGCTCAG -3′) in the hypervariable region of 18S rDNA for PCR (Lin et al., 2012). PCRs were performed in a 25 μl reaction, which contained 12.5 μl 2 × KAPA HiFi HotStart ReadyMix, 0.25 μmol L^-1^ of each primer and 10 ng of DNA template. Thermocycling conditions included starting denaturation at 95°C for 3 min, 25 cycles at 95°C for 30 s, Tm for 30 s, 72°C for 30 s, and 72°C for 5 min. In process of the amplification reaction, the indexes allowing sample multiplexing during sequencing were integrated between the Illumina Miseq adaptor and the reverse primer. The PCR products for each specimen were combined to prepare the PCR amplicon libraries. The PCR products were quantified by applying the Agilent 2100 Bioanalyzer System (Santa Clara, CA, United States) after purification and they were then combined at equal concentrations. Amplicon sequencing was conducted based on the Illumina Miseq platform at Beijing Ori-Gene Science and Technology Co., Ltd. (Beijing, China). The PCR product was purified using Ampure XP beads. In addition, the PCR product was recovered using a QIAquick Gel Extraction kit.

### Processing of Sequencing Data

The 18S data were purified as follows: (1) We removed sequences with sequencing quality scores less than 20, removed sequences containing N, and removed sequences of >10 bp; (2) We removed sequences with primer mismatch (>4 bp) and; (3) removed primer sequences except for short (less than 200 bp) and overly long (>500 bp) sequences; (4) We used UCHIME software as a reference to remove chimeras from the height abundance sequence (Schloss et al., 2009). The sequence was loaded into OTUs (operational taxonomic units) to which the 97% identity was applied. Then, representative sequences were selected according to the most abundant sequence in each OTU. We deposited the raw sequence data into the NCBI Sequence Read Archive database with accession number SRP150029.

The Chao 1 and Shannon indexes were determined, and principal component analysis (PCA) was performed using the UniFrac distance matrix (Lozupone and Knight, 2005). The rarefaction curves of the Chao 1 and Shannon indexes were applied to compare the fungal diversity and richness in the different tillage systems. PCA was performed based on the sequences and OTUs obtained using R 3.12 software. LEfSe software was used to analyze significant differences between the microorganisms in different treatments.

### Analysis of Soil Properties

All three replicates were used in the analysis of soil chemistry characteristics. The SOCs in bulk soil and aggregations were determined using the dichromate oxidation method (Bao, 2000). Soil aggregates were divided using a dry-sieving method based on Huang et al. (2007). Soil aggregates with diameters >2 mm (large macro-aggregates), 2–0.25 mm (small macro-aggregates), 0.053–0.25 mm (micro-aggregates) and <0.053 mm (silt and clay fractions) were separated by shaking the sieves mechanically with an amplitude 1.5 mm for 2 min. The mean weight diameter (MWD) was applied to represent soil aggregate stability and was calculated according to He et al. (2018):

(1) $$MWD=\sum_{1}^{n+1}\frac{r_{i-1}+r_{i}}{2}\times m_{i}$$

where MWD is the mean weight diameter (mm), *r_i_* is the diameter of each part class (mm), and *m_i_* is the weight proportion of soil aggregates compared to the total weight in each class.

The differences in the percentage of soil aggregate contents, their associated C contents, and MWD between tillage treatments, were tested using SPSS 17.0.

## Results

### Impact of Tillage Regimes on Soil Aggregates Distribution

**FIGURE 1:**
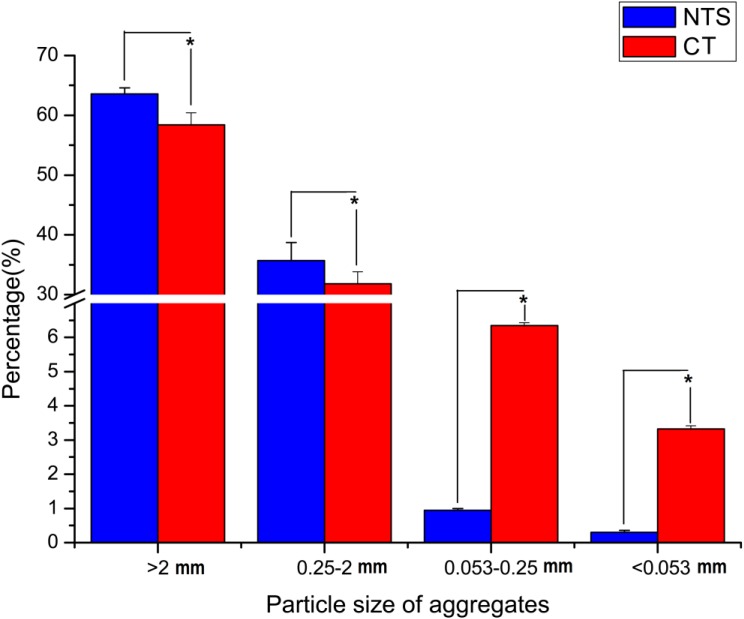
Effect of tillage treatments on soil aggregates size distributions. ^∗^*p* < 0.05.

**FIGURE 2:**
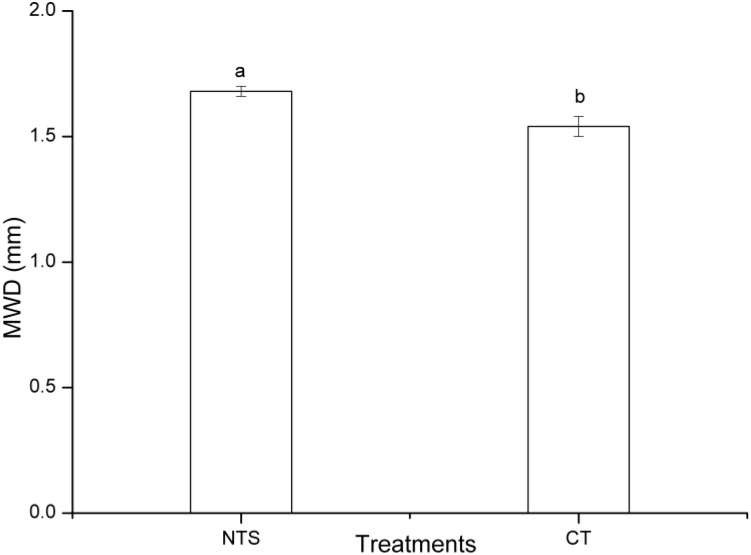
The MWD (mean weight diameter) under no tillage with crop straw returning (NTS) and conventional tillage without the crop straw (CT) treatments. Different letters above the standard error bars indicate significant differences between treatments at the *p* < 0.05 level.

The results showed that different tillage treatments significantly (*P* < 0.05) changed the distribution of soil aggregates. Soil aggregates were mainly composed of large aggregates. However, the percentage of large macro-aggregates was significantly (*P* < 0.05) greater in NTS, being 9.1% (>2 mm) and 12.2% (0.25–2 mm) higher than in the CT treatment, respectively (Figure 1 and Supplementary Table S1). Meanwhile, NTS treatment significantly (*P* < 0.05) increased the MWD by 9.8% as compared with CT treatment (Figure 2 and Supplementary Table S2). However, NTS significantly (*P* < 0.05) decreased the proportions of micro-aggregates (0.053–0.25 mm), and silt and clay fractions (<0.053 mm) by 88.1 and 92.6%, respectively (Figure 1 and Supplementary Table S1).

### Effect of Tillage on SOC in Aggregates

**FIGURE 3:**
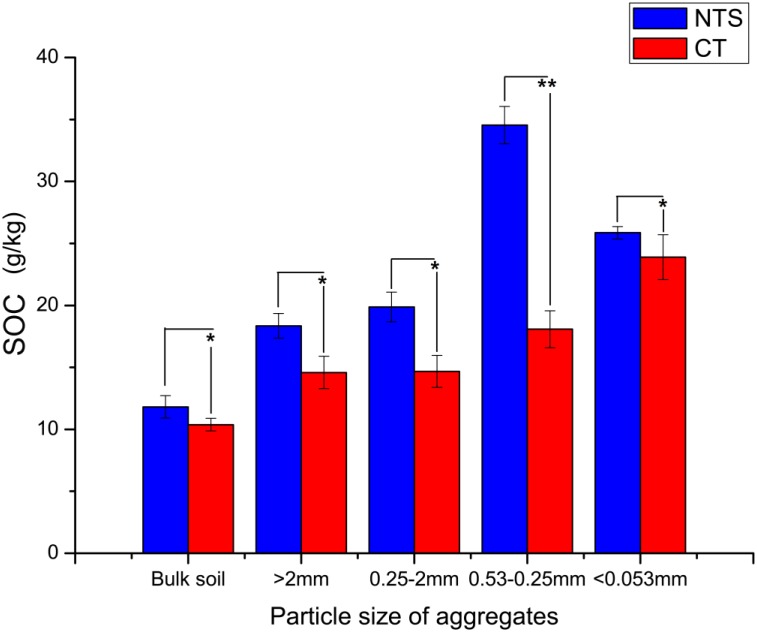
The SOC (soil organic carbon content) in bulk soil and its aggregations under no tillage with crop straw returning (NTS) and conventional tillage without the crop straw (CT) treatments. ^∗^*p* < 0.05 and ^∗∗^*p* < 0.01.

As shown in Figure 3, NTS soil had higher SOC in all aggregates and bulk soil than those of CT treatment. Compared to the CT treatment, SOC in the large macro-aggregates, small macro-aggregates, micro-aggregates, and silt and clay parts was 25.2, 35.1, 91.1, and 9.2% greater, respectively. Moreover, NTS treatment significantly increased SOC in bulk soil by 10.0% as compared with CT (Figure 3 and Supplementary Table S3). All the above results show that NTS treatment can significantly improve the physical and chemical characteristics of soil.

### Analysis of Rarefaction Curves

**FIGURE 4:**
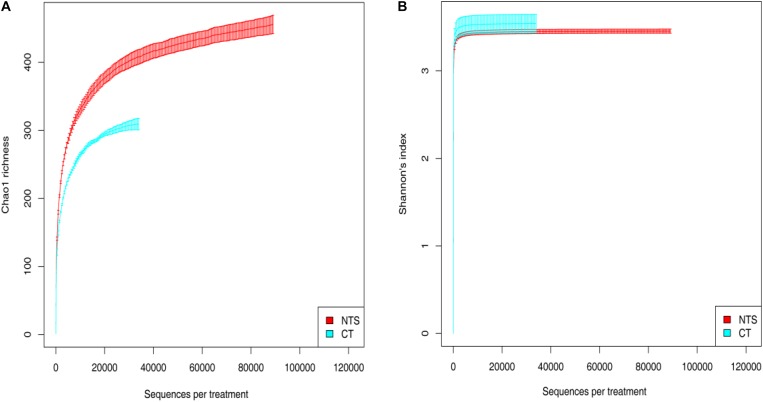
Rarefaction curves of Chao 1 **(A)**, and Shannon index **(B)** for no tillage with crop straw returning (NTS) and conventional tillage without the crop straw (CT) treatments.

The Chao1 and Shannon indexes were used to reflect species diversity. The greater their values, the higher the species diversity of the sample. The rarefaction curves of the Chao 1 and Shannon indexes in different tillage systems are shown in Figure 4. When the number of sequences exceeded 1000, all amplified dilution curves reached a plateau, which meant that the sequence-derived diversity and abundance assessed in the present study could sufficiently characterize the fungal species in each sample (Figure 4). Alpha diversity analysis suggested that there was a significant difference in the richness index (Chao1) between the NTS and CT treatments. The Chao 1 value of the NTS treatment was significantly higher than that of the CT treatment. The results indicate that NTS treatment is beneficial to fungi.

### Overall Taxonomic Information

**FIGURE 5:**
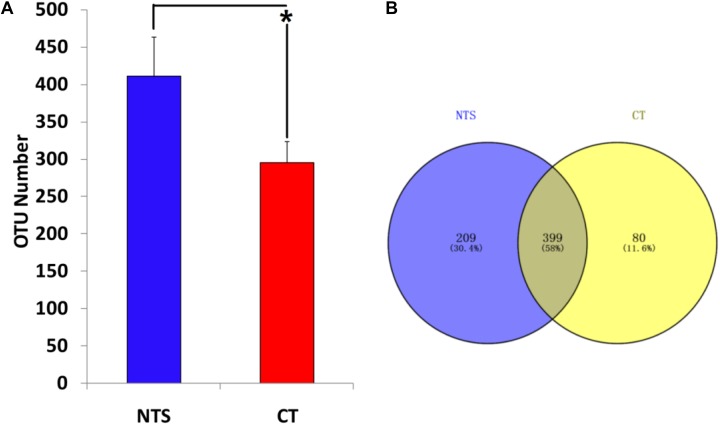
The OTU number **(A)** and venn diagram **(B)** of soil Arbuscular mycorrhizal (AM) fungi under no tillage with crop straw return (NTS) and conventional tillage without the crop straw (CT) treatments. ^∗^*p* < 0.05.

A total of 507,263 quality sequences were obtained by quality control analyses of the raw data. According to the 97% species similarity level, a total of 688 OTUs were derived in this study. The results show that the OTU number of the NTS treatment was significantly higher than that of the CT treatment (Figure 5). Moreover, the Venn diagram intuitively indicates the common and special OTUs. Venn analysis showed that only 58% of OTUs (399) were shared by the different treatments. There were 209 and 80 particular OTUs in the NTS and CT treatments, respectively (Figure 5).

**FIGURE 6:**
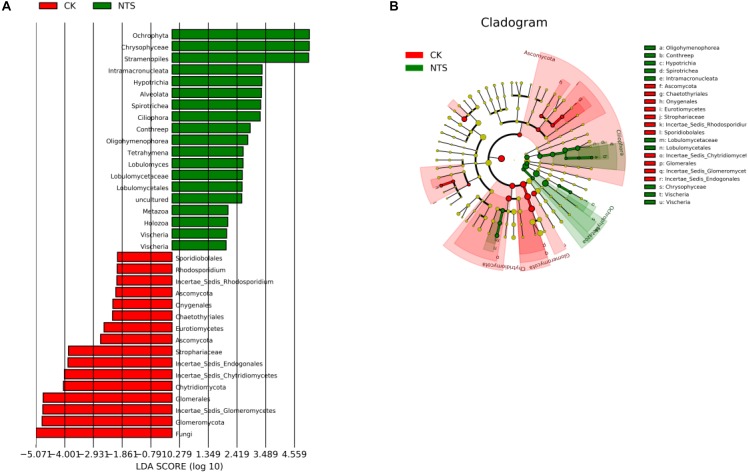
The differences in the LDA (Linear Discriminant Analysis) distribution histogram **(A)** and evolutionary branch diagram **(B)** of special microorganisms under two tillage treatments: no-tillage with crop straw return (NTS) and conventional tillage without crop straw (CT).

To further analyze the differences between species, LEfSe software was used to determine significant differences in the fungal species present in the different treatments. The results showed that the relative abundances of *Oligohymenophorea, Conthreep, Hypotrichia, Spirotrichea, Intramacronucleata, Lobulomycetaceae, Lobulomycetales, Chrysophyceae*, and *Vischeria* were significantly increased in the NTS treatment, while the relative abundances of *Ascomycota, Chaetothyriales, Onygenales, Eurotiomycetes, Strophariaceae, Incertae Sedis Rhodosporidium, Sporidiobolales, Incertae Sedis Chytridiomycetes, Glomerales, Incertae Sedis Glomeromycetes*, and *Incertae Sedis Endogonales* were significantly decreased in the NTS treatment (Figure 6). These results indicate that there is a close correlation between the soil tillage method and the composition of microorganisms.

### Composition of AM Fungi Under Different Tillage Treatments

**FIGURE 7:**
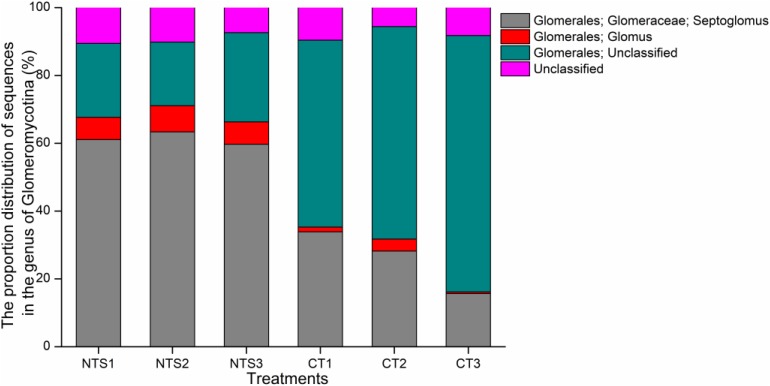
The proportion of sequences in *Glomeromycotina* under no tillage with crop straw return (NTS) and conventional tillage without the crop straw (CT) treatments.

Arbuscular mycorrhizal fungi are common fungal species in soil and play a key role in plant growth and plant resistance to plant disease. We also analyzed the species composition of AM fungi. For different tillage treatments, the *Glomeromycotina* sequences varied in number from 4551 to 47,055, becoming 4 to 29 of the OTUs, respectively. The sequence proportions of the different *Glomeromycotina* species are shown in Figure 7. The NTS treatment had higher proportions of *Septoglomus* and *Glomus* than CT. The proportions of *Glomeromycetes* varied in the two treatments (Supplementary Table S4).

### Relationship Between Soil AM Fungal Communities and Aggregate Contents

**FIGURE 8:**
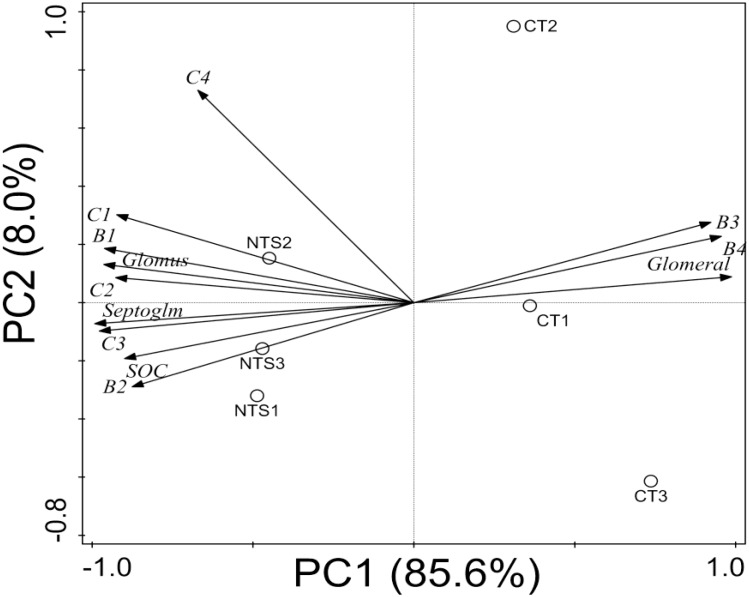
Biplot of principal components PC1 and PC2 from all soil samples and variables: percentage of soil large macro-aggregates (B1); percentage of small macro-aggregates (B2); percentage of micro-aggregates (B3); percentage of the silt and clay fraction (B4); SOC in large macro-aggregates (C1); SOC in small macro-aggregates (C2); SOC in micro-aggregates (C3); SOC in the silt and clay parts (C4); mean weight diameter (MWD); percentage of *Septoglomus* (*Septoglm*); percentage of *Glomus* (*Glomus*); percentage of *Glomerales* unclassified (*Glomeral*) sequences for the following cases: no-tillage with crop straw (NTS) and conventional tillage without crop straw (CT), based on correlations of the 12 variables specified in Table 1.

Pearson correlation coefficients and PCA were applied to evaluate the relationship between soil AM fungal communities (i.e., the percentages of *Septoglomus, Glomus*, and *Glomerales* unclassified) and soil physical and chemical properties (i.e., SOC in bulk soil, soil aggregates and their associated C contents). The PCA (Figure 8) showed that that the first component explained 85.6% of the total variance. Moreover, it was negatively associated with the percentage of large and small macro-aggregates and the SOCs in macro- and micro-aggregates and the silt and clay parts, the proportions of *Glomus*, and *Septoglomus*. The second component of the PCA explained 8.0% of the overall variance and was positively correlated with the percentage of large macro-aggregates, the SOCs in macro-aggregates and the silt and clay fraction, the percentage of *Glomus*.

**Table 1:** Pearson correlation coefficients between the soil properties and percentages of sequences in *Glomeromycotina*.

	*Septoglomus*	*Glomus*	*Glomerales*	B1	B2	B3	B4	C1	C2	C3	C4	SOC
*Septoglomus*	1.000	0.927^**^	-0.999^**^	0.942^**^	0.921^**^	-0.914^*^	-0.950^**^	0.907^*^	0.900^*^	0.973^**^	0.614ns	0.903^*^
*Glomus*		1.000	-0.923^**^	0.978^**^	0.750ns	-0.885^*^	-0.910^*^	0.896^*^	0.872^*^	0.926^**^	0.743ns	0.867^*^
*Glomerales*			1.000	-0.943^**^	-0.921^**^	0.914^*^	0.950^**^	-0.902^*^	-0.879^*^	-0.971^**^	-0.596ns	-0.899^*^
B1				1.000	0.795ns	-0.825^*^	-0.867^*^	0.928^**^	0.836^*^	0.906^*^	0.786ns	0.864^*^
B2					1.000	-0.786ns	-0.832^*^	0.716ns	0.762ns	0.834^*^	0.410ns	0.923^**^
B3						1.000	0.995^**^	-0.779ns	-0.860^*^	-0.968^**^	-0.394ns	-0.820^*^
B4							1.000	-0.824^*^	-0.887^*^	-0.985^**^	-0.457ns	-0.853^*^
C1								1.000	0.893^*^	0.906^*^	0.818^*^	0.691ns
C2									1.000	0.920^**^	0.683ns	0.773ns
C3										1.000	0.562ns	0.826^*^
C4											1.000	0.510ns
SOC												1.000


*ns, there was no significant difference. ^∗^*p* < 0.05, ^∗∗^*p* < 0.01. B1, percentage of soil large macro-aggregates; B2, percentage of small macro-aggregates; B3, percentage of micro-aggregates; B4, percentage of the silt and clay parts; C1, SOC in large macro-aggregates; C2, SOC in small macro-aggregates; C3, SOC in micro-aggregates; C4, SOC in the silt and clay fraction; SOC, SOC in bulk soil; *Septoglomus*, percentage of *Septoglomus*; *Glomus*, percentage of *Glomus*; *Glomerales*, percentage of *Glomerales* unclassified.*

Moreover, the PCA and Pearson correlation coefficient results showed that the proportion of *Septoglomus* was positively associated with that of *Glomus*, the percentages of soil macro-aggregates and their associated C content, the SOC in micro-aggregates, and the SOC in bulk soil. The proportion of *Septoglomus* was negatively correlated with that of *Glomerales* unclassified, the percentages of micro-aggregates, and the silt and clay fraction. Similarly, the percentage of large macro-aggregates, and the SOC in bulk soil and macro- and micro-aggregates, also are important contributors to the proportion of *Glomus*. In addition to these relationships, the percentage of large macro-aggregates was positively correlated with bulk soil and macro- and micro-aggregates SOC.

In addition, a highly significant multiple linear regression equation was obtained for the purposes of predicting the main influences on bulk SOC (*P* < 0.01, *R*^2^ = 0.85, standard error of the estimate = 0.078; factors considered redundant or irrelevant were excluded from the model according to the step-wise method; Table 2). The results indicate that the percentage of large aggregates was the determining factor of bulk SOC in this region.

**Table 2:** Multiple linear regression models for SOC under different tillage regimes.

Variables	Estimated parameters	SE	*P*-value	*R*^2^
Intercept	-1.420	2.637		
B1	0.374	0.078	0.009	0.851


*B1, intercept parameters; SE, Standard error.*

## Discussion

### Soil Aggregates and Their Associated C Contents

This study indicates that, after 7 years of winter wheat-summer maize rotation, NTS treatment improved the MWD, the percentages of large and small macro-aggregates compared to CT treatment. Similarly, previous studies have also reported that tillage treatment decreased soil macro-aggregates and soil stability, which might be due to the mechanical disruption of macro-aggregates caused by frequent tillage treatments (Bottinelli et al., 2017; Somasundaram et al., 2018). The mean aggregates size is known to decrease as tillage is intensified (Sheehy et al., 2015). When compared to conventionally tillage treatment, no tillage with crop straw return could enhance soil structural stability, this might be due to the increased bulk SOC concentration in the semi-arid and semi-humid area of North China (Du et al., 2017).

Moreover, our results showed that NTS had the higher SOC in all aggregates than those of CT treatment. Tillage management has been found to cause measurable changes in the SOC contents of organic-mineral fractions (Sun et al., 2013). Similarly, zero tillage could reduce the damage to soil aggregates and enhance the concentration and stability of related SOC, which resulted in higher SOC under NTS in comparison with CT treatment (Song et al., 2016). Our results show that NTS treatment could effectively increase SOC in bulk soil mainly by enhancing the percentage of large macro-aggregates as compared with CT, which might be useful to alleviate the greenhouse effect to some extent.

### Composition of AM Fungi

Soil fungal diversity analysis showed that NTS treatment could significantly improve soil microbial composition and diversity. No difference in maize yield was observed between NTS and CT treatments in the present study (Supplementary Table S5). Similarly, other previous studies also showed that tillage methods could influence soil microbial activity and structure by altering the habitat of soil microbes, such as the soil’s gas permeability, soil texture and microbial substrates (Wang et al., 2017), thus affecting SOC. In addition, tillage could change the soil physical structure, strongly undermine the underground mycelium of soil mycorrhizal fungi, and reduce their extension range, infection rate, and community diversity (Anderson et al., 1987; Kabir, 2005). Similarly, other previous studies have also reported that no-till treatment could improve the abundance and the diversity of soil AM fungi as compared to plowed plots, which ultimately improved the plant growth (Boddington and Dodd, 2000; Wetzel et al., 2014; Hu et al., 2015). Moreover, our results showed that the percentage of macro-aggregates, its associated C content, SOCs in small macro-aggregates and micro-aggregates, and SOC in bulk soil were related with the percentage of *Septoglomus*. Similarly, Qin et al. (2017) also showed that the percentage of macro-aggregates (0.25–2 mm) was positively related with soil AM fungi biomass.

However, other studies showed that long-term no-till treatment could decrease the soil AM fungal propagules because of the higher soil bulk density, and the lower C utilization efficiency of soil organisms as compared with the plowed plots (Fu et al., 2000; Curaqueo et al., 2011; Schluter et al., 2018). These inconsistent conclusions may be due to the differences in soil properties, climatic conditions and the duration of no-till treatment studied. In addition, we also found some significant differences in the soil fungal communities of the two treatments that might have a close relationship with plant growth and the physical and chemical characteristics of soil. Our results suggested that the effect of tillage treatment on AM fungi community might be more important at a long-time scale. Thus, it is a need for a long-term study to focus on the effect of various tillage treatments on AM fungi community and its relationship with crop growth and soil properties (i.e., soil physical and chemical characteristics) during crop growth on Loess Plateau in China.

## Conclusion

In this study, tillage treatment changed soil aggregate distributions and their associated C contents, with the NTS treatment having more macro-aggregates and associated C contents than the CT treatment. Meanwhile, NTS treatment significantly increased the percentages of *Septoglomus* and *Glomus* compared with CT treatment. We also found some significant differences in soil fungal communities between the two treatments. In addition, Pearson correlation coefficients and PCA identified a close relationship between SOC levels and the proportions of *Septoglomus* and *Glomus* in the soil community. Step-wise regression analysis indicated that NTS promoted SOC at the surface soil layer (0–20 cm), probably by enhancing the percentage of large macro-aggregates therein. Above all, our results indicate that NTS conditions favor the maintenance of AM fungi, soil structure and SOC and might play a key role in the development of agricultural sustainability in the Loess Plateau of China. This long-term study was based on a 7-year field trial and provides insights into the consequences of agricultural practices on soil properties and microorganisms, thereby playing a role in agricultural sustainability. However, further study is needed to investigate the relationships between soil AM communities, soil physical and chemical characteristics, and crop production in the Loess Plateau, China.

## Author Contributions

XlL and YL designed the experiments. XlL and XnL carried out the experiments and performed the analyses. XlL contributed to writing the paper. YL checked the paper.

## Conflict of Interest Statement

The authors declare that the research was conducted in the absence of any commercial or financial relationships that could be construed as a potential conflict of interest.
