# Supplementary material for: Effect of Tillage Treatment on the Diversity of Soil Arbuscular Mycorrhizal Fungal and Soil Aggregate-Associated Carbon Content
Source: Front Microbiol. 2018 Dec 6;9:2986. doi: 10.3389/fmicb.2018.02986 (PMC6291503; doi:10.3389/fmicb.2018.02986)
Supplement: Supplementary file 1 [file Table_1.DOCX]

**Table S1.** The percentage of soil aggregates size distribution (%) under different tillage treatments.

| **Treatments** | **>2 mm** | **0.25-2 mm** | **0.053-0.25 mm** | **<0.053 mm** |
| --- | --- | --- | --- | --- |
| NTS | 63.6±0.56a | 35.58±0.56a | 0.76±0.09b | 0.25±0.03b |
| CT | 58.29±1.35b | 31.72±1.10b | 6.37±0.34a | 3.38±0.03a |

NTS, No tillage with straw returning; CT, conventional moldboard plowing tillage without straw. The values represent the means±standard errors. The different lower case letters following the numbers indicate the difference between tillage treatments at 5% significance levels.
