# Supplementary material for: Effect of Tillage Treatment on the Diversity of Soil Arbuscular Mycorrhizal Fungal and Soil Aggregate-Associated Carbon Content
Source: Front Microbiol. 2018 Dec 6;9:2986. doi: 10.3389/fmicb.2018.02986 (PMC6291503; doi:10.3389/fmicb.2018.02986)
Supplement: Supplementary file 2 [file Table_2.docx]

**Table S2.** The mean weight diameter (mm) under different tillage treatments.

| **Treatments** | **Mean weight diameter (mm)** |
| --- | --- |
| NTS | 1.68±0.02a |
| CT | 1.54±0.04b |

NTS, No tillage with straw returning; CT, conventional moldboard plowing tillage without straw. The values represent the means±standard errors. The different lower case letters following the numbers indicate the difference between tillage treatments at 5% significance levels.
