# Supplementary material for: Effect of Tillage Treatment on the Diversity of Soil Arbuscular Mycorrhizal Fungal and Soil Aggregate-Associated Carbon Content
Source: Front Microbiol. 2018 Dec 6;9:2986. doi: 10.3389/fmicb.2018.02986 (PMC6291503; doi:10.3389/fmicb.2018.02986)
Supplement: Supplementary file 3 [file Table_3.docx]

**Table S3.** The soil organic carbon content (g kg^-1^) in all aggregates under different tillage treatments.

| **Treatments** | **Bulk soil** | **>2 mm** | **0.25-2 mm** | **0.053-0.25 mm** | **<0.053 mm** |
| --- | --- | --- | --- | --- | --- |
| NTS | 11.33±0.43a | 18.3±0.62a | 19.89±1.o6a | 34.53±0.97a | 25.9±0.21a |
| CT | 10.3±0.43b | 14.62±1.08b | 14.72±0.84b | 18.07±0.99b | 23.72±2.05b |

NTS, No tillage with straw returning; CT, conventional moldboard plowing tillage without straw. The values represent the means±standard errors. The different lower case letters following the numbers indicate the difference between tillage treatments at 5% significance levels.
