# Supplementary material for: Effect of Tillage Treatment on the Diversity of Soil Arbuscular Mycorrhizal Fungal and Soil Aggregate-Associated Carbon Content
Source: Front Microbiol. 2018 Dec 6;9:2986. doi: 10.3389/fmicb.2018.02986 (PMC6291503; doi:10.3389/fmicb.2018.02986)
Supplement: Supplementary file 4 [file Table_4.DOCX]

**Table S4.** The percentage of sequences in *Glomeromycotina* (%) under different tillage treatments.

| **Treatments** | **Septoglomus** | **Glomus** | **Glomerales Unclassified** | **Unclassified** |
| --- | --- | --- | --- | --- |
| NTS | 61.4±1.06a | 6.96±0.38a | 22.28±2.18b | 9.36±0.99a |
| CT | 25.93±5.38b | 1.80±0.90b | 64.47±5.97a | 7.8±1.17a |

NTS, No tillage with straw returning; CT, conventional moldboard plowing tillage without straw. The values represent the means±standard errors. The different lower case letters following the numbers indicate the difference between tillage treatments at 5% significance levels.
