# Supplementary material for: Effect of Tillage Treatment on the Diversity of Soil Arbuscular Mycorrhizal Fungal and Soil Aggregate-Associated Carbon Content
Source: Front Microbiol. 2018 Dec 6;9:2986. doi: 10.3389/fmicb.2018.02986 (PMC6291503; doi:10.3389/fmicb.2018.02986)
Supplement: Supplementary file 5 [file Table_5.DOCX]

**Table S5.** The maize yield (t ha^-1^) under NTS and CT treatments.

| **Treatments** | **2014-15** |
| --- | --- |
| NTS | 8.27±0.46a |
| CT | 7.97±0.42a |

NTS, No tillage with straw returning; CT, conventional moldboard plowing tillage without straw. The values represent the means±standard errors. The same lower case letters following the numbers indicate no difference between tillage treatments at 5% significance levels.
